# Analysis of global gene expression profile of rice in response to methylglyoxal indicates its possible role as a stress signal molecule

**DOI:** 10.3389/fpls.2015.00682

**Published:** 2015-09-03

**Authors:** Charanpreet Kaur, Hemant R. Kushwaha, Ananda Mustafiz, Ashwani Pareek, Sudhir K. Sopory, Sneh L. Singla-Pareek

**Affiliations:** ^1^Plant Molecular Biology Group, International Centre for Genetic Engineering and BiotechnologyNew Delhi, India; ^2^Synthetic Biology and Biofuels Group, International Centre for Genetic Engineering and BiotechnologyNew Delhi, India; ^3^Stress Physiology and Molecular Biology Laboratory, School of Life Sciences, Jawaharlal Nehru UniversityNew Delhi, India

**Keywords:** methylglyoxal, rice, signal transduction, signaling, transcriptome

## Abstract

Methylglyoxal (MG) is a toxic metabolite produced primarily as a byproduct of glycolysis. Being a potent glycating agent, it can readily bind macromolecules like DNA, RNA, or proteins, modulating their expression and activity. In plants, despite the known inhibitory effects of MG on growth and development, still limited information is available about the molecular mechanisms and response pathways elicited upon elevation in MG levels. To gain insight into the molecular basis of MG response, we have investigated changes in global gene expression profiles in rice upon exposure to exogenous MG using GeneChip microarrays. Initially, growth of rice seedlings was monitored in response to increasing MG concentrations which could retard plant growth in a dose-dependent manner. Upon exposure to 10 mM concentration of MG, a total of 1685 probe sets were up- or down-regulated by more than 1.5-fold in shoot tissues within 16 h. These were classified into 10 functional categories. The genes involved in signal transduction such as, protein kinases and transcription factors, were significantly over-represented in the perturbed transcriptome, of which several are known to be involved in abiotic and biotic stress response indicating a cross-talk between MG-responsive and stress-responsive signal transduction pathways. Through *in silico* studies, we could predict 7–8 bp long conserved motif as a possible MG-responsive element (MGRE) in the 1 kb upstream region of genes that were more than 10-fold up- or down-regulated in the analysis. Since several perturbations were found in signaling cascades in response to MG, we hereby suggest that it plays an important role in signal transduction probably acting as a stress signal molecule.

## Introduction

Methylglyoxal (MG) is a highly reactive cytotoxic α-oxoaldehyde that is formed endogenously via both enzymatic and non enzymatic reactions, mainly as a byproduct of glycolysis from triose phosphates. In addition, MG is also generated as a side-product of amino acid and acetone metabolism (Kalapos, 1999). At higher than physiological levels, MG inhibits cell proliferation (Ray et al., 1994) which is likely due to its ability to readily react with and modify macromolecules such as DNA, RNA, and proteins, forming advanced glycation end products (AGEs) (Thornalley, 2008). In animals, MG-mediated post-translational protein modifications are believed to be one of the causative factors of aging as well as of a number of diseases, including cancer and diabetes (Ramasamy et al., 2006). MG levels also rise to toxic concentrations in plants on exposure to abiotic and biotic stresses. Its concentration in rice at physiological conditions is ~2 μmol/g fresh weight and rises two- to six-fold in response to salinity, drought, and cold conditions (Yadav et al., 2005a; Ghosh et al., 2014). To keep its levels under control, several detoxification mechanisms exist in living systems. The glutathione (GSH)-dependent glyoxalase system comprising glyoxalase I and glyoxalase II enzymes, is believed to be the major detoxification route for MG, converting it to D-lactate. Other enzymes involved in MG detoxification include GSH-independent glyoxalase III enzyme, aldo-keto reductase, and dehydrogenases (Kalapos, 1999).

Interestingly, despite its adverse effects on cell growth, *E. coli*, and other microorganisms possess enzymes which generate MG through catalytic reactions. MG synthase is one such enzyme which converts dihydroxyacetone phosphate, a glycolytic intermediate (Ferguson et al., 1998) to MG and inorganic phosphate. When MG is so toxic, its catalytic synthesis raises questions regarding its physiological role in the living systems. It is however proposed that in bacteria, MG production might allow cells to control carbon influx in case an imbalance in metabolism occurs (Booth et al., 2003). Also, MG is known to induce Ca^2+^ transients in *E. coli*; apparently by opening Ca^2+^ channels in a dose-dependent manner (Campbell et al., 2007). This suggests an important role for this metabolite in bacterial-host cell signaling. Likewise in yeast, MG has been shown to act as a signal initiator during oxidative stress (Maeta et al., 2005). On the other hand, the probable role of MG as a signal molecule in plants is yet to be examined. Nonetheless, some indications can be taken from the previous reports which demonstrate that MG is never completely depleted from the system, maintaining a threshold level under normal growth conditions (Singla-Pareek et al., 2003, 2006; Yadav et al., 2005a,b; Hossain et al., 2009), pointing toward its potential role in signal transduction.

In order to understand the physiological and molecular details underlying MG response, in the present study, we have investigated changes in rice transcriptome upon exogenous application of MG. We analyzed physiological changes in MG-exposed rice seedlings followed by a whole genome microarray analysis. Several genes were found to be up- and down-regulated upon MG application. Interestingly, we observed a significant alteration in genes involved in signal transduction. Further, analysis of upstream sequences of MG-responsive genes (up- and down-regulated genes) led to the identification of few conserved motifs which may serve as MG-response element (MGRE). Overall, the present study strongly indicates an important role of MG in signal transduction and provides first information on global expression profile of MG-responsive transcriptome in plants, suggesting diverse biological functions of MG in plants.

## Material and methods

### Plant material and growth conditions

Plants of rice cultivar IR64 were grown in controlled conditions in growth chamber at 28 ± 2°C and 16 h photoperiod. The seeds were surface sterilized with 1% Bavistin for 20 min and allowed to germinate in a hydroponics culture system. Germinated seeds were supplied with the modified Yoshida medium (Yoshida et al., 1972).

### Morphological analysis

For seedling growth experiments, 7 day old seedlings of IR64 rice were transferred to Yoshida medium supplemented with different concentrations of MG (5, 7.5, 10, 15, and 20 mM) for 16 h. Untreated seedlings were used as control. After 16 h, seedlings were rescued from MG medium and transferred to Yoshida medium for recovery. Growth of the seedlings was monitored for 4 days after recovery and root and shoot length were measured from control and MG treatment to assess plant growth.

### RNA extraction and GeneChip microarray experiment

IR64 rice cultivar was chosen for microarray analysis. Seven day old seedlings were treated with 10 mM MG for 16 h in hydroponics. Shoot tissue was harvested and total RNA was isolated from the control and MG-treated samples as described previously (Kaur et al., 2013). RNA samples were used for Affymetrix rice chip hybridization experiment and data analysis. A total of four hybridizations were carried out as two biological replicates for each condition (control and MG treatment). Single color Affymetrix rice chip was used, where only one dye (Cy3 dye) was used per sample/per hybridization and the arrays were processed further as per the GeneChip microarray (Affymetrix) manufacturer's protocol. Quality control analysis was carried out prior to cDNA synthesis followed by its labeling and hybridization and the expression data of 57,381 probe sets was generated. Overall quality of the prepared hybridized chip was assessed using sample correlation and principle component analysis (PCA) (Table S1 and Figure S1).

### Microarray data analysis

The Robust Multiarray Average (RMA) algorithm was used for normalization and probe summarization. The resultant normalized expression values were log_2_ transformed and utilized for further analysis. In order to reduce false positives which can arise due to poor reproducibility among the replicates, we used statistical stringency of *p*≤0.05.

Linear modeling approach was used for the assessment of differential expression. The limma library from R package was used to construct linear models with arbitrary coefficients and contrasts of interest. To obtain differentially expressed genes, moderated t-statistic has been implemented. The multiplicity of testing was performed using Benjamin and Hochberg (BH) correction adjusting for the false discovery rate (FDR). A threshold adjusted *p*-value was set to 0.05, and the fold-change threshold was set to 1.5. Only the transcripts with a minimum 1.5-fold increase or decrease in signal over the control were identified as “MG-responsive” genes. The functional categories were obtained from the annotation of the probes using NetAffx software developed for Affymetrix microarray chips. Further, clustering analysis was performed within conditions using Euclidean distance metric and Centroid linkage rule (Figure S2).

### Prediction of MG-responsive motifs

For the identification of probable MG-responsive *cis*-regulatory elements in the promoter region of genes being 10-fold or more induced or repressed upon MG treatment, we downloaded 1 kb sequences upstream of ATG initiation codon of “MG-responsive” genes from the Rice genome annotation project database (http://rice.plantbiology.msu.edu/index.shtml). The conserved motifs were predicted using the Multiple Expectation for Motif Elicitation (MEME) software (Bailey et al., 2009) and validated *in silico* across the members of glyoxalase family reported in Mustafiz et al. (2011).

## Results

### Exogenously supplied methylglyoxal inhibits growth of rice seedlings

**Figure 1:**
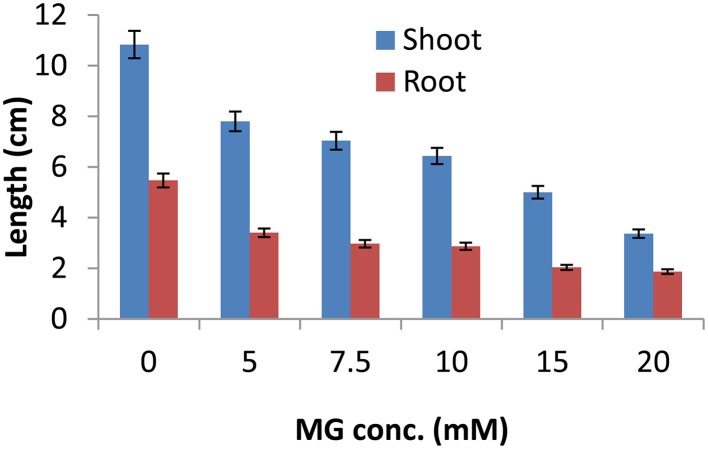
**Methylglyoxal inhibits growth of rice seedlings**. One week-old IR64 rice seedlings were subjected to various concentrations of MG (0, 5, 7.5, 10, 15, and 20 mM) for 16 h and assessment of growth parameters (shoot and root length) was done after 4 days of recovery.

To study the effect of exogenous application of MG on growth of rice plants, 7 day old seedlings of IR64, a high yielding but salt-sensitive cultivar of rice were subjected to different concentrations (0, 5, 7.5, 10, 15, and 20 mM) of MG for 16 h. After 16 h, seedlings were allowed to recover for 4 days following which root and shoot length was measured to determine the effect of MG application on plant growth. MG exposure adversely affected growth of rice seedlings with profound effect on root elongation (Figure 1). Upto 10 mM MG concentration, MG exposure caused reduction in growth of rice seedlings in a dose-dependent manner. But at higher than 10 mM concentration, growth inhibition was observed to be very severe causing more than 50% decrease in both root and shoot length (Figure 1). Thus, MG could be clearly seen to affect the growth of rice seedlings with severe retardation in growth of plants exposed to concentrations greater than 10 mM MG. Hence, for subsequent analysis, 10 mM MG concentration was used.

### MG causes a global change in rice gene expression profile

Gene expression profiles were determined using 1 week-old IR64 rice seedlings exposed to 10 mM MG for 16 h. Detailed analysis of the transcriptome profile obtained through microarray experiment revealed several intriguing facts. The rice transcriptome showed a global alteration upon MG exposure. Using an Affymetrix GeneChip microarray containing 57,381 probe sets, we identified 1685 probe sets which displayed greater than 1.5-fold change in expression upon application of exogenous MG compared to the untreated control. Of these, 719 probe sets were down-regulated and 966 were up-regulated. While main emphasis is usually given to the identification of the up-regulated genes, the down-regulation of gene expression also contributes to the adaptation of plants to a given stimuli. A reasonable assumption is that genes are down-regulated because their product may not be suited to the new physiological conditions caused by the external stimuli (Chandler and Robertson, 1994). A *ProDH* gene is down-regulated during water stress, leading to the accumulation of proline which helps in restoring osmotic balance during stress (Yoshiba et al., 1997). Also, down-regulation of some genes may be required for reprogramming of protein synthesis under stress such as, a 60S ribosomal protein L32 encoding gene, *rpL32*, is down-regulated at the transcriptional level under abiotic stress through the removal of transcription factors from the *cis*-elements in its promoter region (Mukhopadhyay et al., 2011) in turn contributing to reprogramming mechanisms.

**Figure 2:**
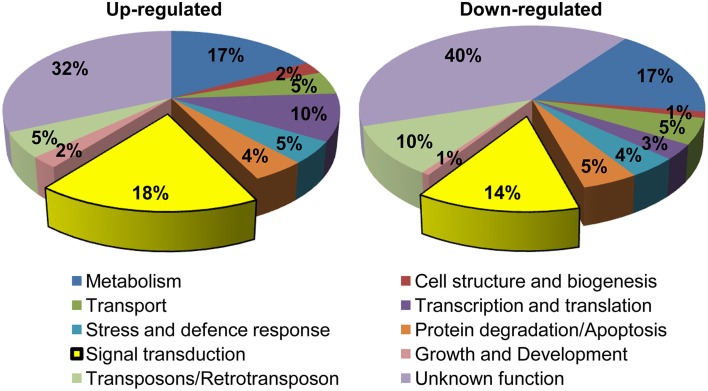
**Functional classification of MG-responsive genes**. Genes showing more than 1.5-fold change in expression were grouped into 10 functional categories. In total, 966 probe sets were up-regulated and 719 were down-regulated in response to MG.

The MG-responsive genes obtained from the microarray experiment could be classified into 10 categories: (1) Signal transduction, (2) Transcription and translation, (3) Transposons/retrotransposons, (4) Transport, (5) Stress and defense response, (6) Metabolism, (7) Cell structure and biogenesis, (8) Protein degradation/apoptosis, (9) Growth and development, and (10) Unknown function (Figure 2). The genes encoding unknown proteins formed the largest category. About 32% of the total up-regulated and 40% of the total down-regulated genes lied in this category (Figure 2). These expressed proteins can open up new avenues for the identification of novel proteins important for MG response. The next largest category belonged to genes involved in metabolism, with 17% of genes being up-regulated and equal percentage being down-regulated upon MG application. This category included genes involved in primary metabolism, i.e., carbohydrate (e.g., hexokinase, triose phosphate isomerase), lipid (e.g., fatty acid hydroxylase, omega-3 fatty acid desaturase), and amino acid (e.g., shikimate kinase, serine hydroxymethyltransferase) metabolism; and also secondary metabolism. An alteration in the metabolic pathways is expected since MG is an inevitable byproduct of glycolysis, a carbohydrate metabolic pathway. A rise in MG concentration possibly acts as a signal for the system to adjust its energy needs through alteration in metabolic pathways as a whole.

Further, genes involved in signal transduction formed another significant category, with 18% of the total genes being up-regulated and 14% being down-regulated indicating a large-scale alteration in signaling pathways upon exposure to exogenous MG. We also observed a change in the expression of around 13% genes involved in transcription and translation processes, which is probably required for reprogramming cellular machinery during adaptation to MG. Notably, 9% of the MG-responsive transcriptome involved genes required for degradation and apoptosis. This is because MG can irreversibly modify proteins at arginine and lysine residues, with arginine having the highest probability among all amino acids for location at functional sites of proteins often leading to functional impairment, and in turn triggering cellular proteolysis (Rabbani and Thornalley, 2014). In this context, a dicarbonyl proteome has been defined which includes proteins susceptible to modifications by MG such as albumin, hemoglobin, transcription factors, mitochondrial proteins, and other proteins linked to mitochondrial dysfunction, oxidative stress, and apoptosis (Rabbani and Thornalley, 2012). Furthermore, several stress and defense responsive genes were also altered possibly as a part of general response to combat adverse conditions. Overall, we could observe a global alteration in rice transcriptome upon MG application.

### Identification of MG-responsive signal transduction pathways

Of all the genes affected by exogenous application of MG, the cluster comprising genes involved in signal transduction formed a significant part (32% of the total up/down regulated genes) (Figure 2). The signal transduction pathway is a complex cascade of genes working in a fine-tuned manner to regulate gene transcription patterns in response to extracellular stimuli. The gene regulatory signals are transmitted through the cytoplasm via protein kinases, which are essential for communication between the cytoplasm and nucleus, and act by carrying out signal-dependent phosphorylation/dephosphorylation of transcription factors.

**Figure 3:**
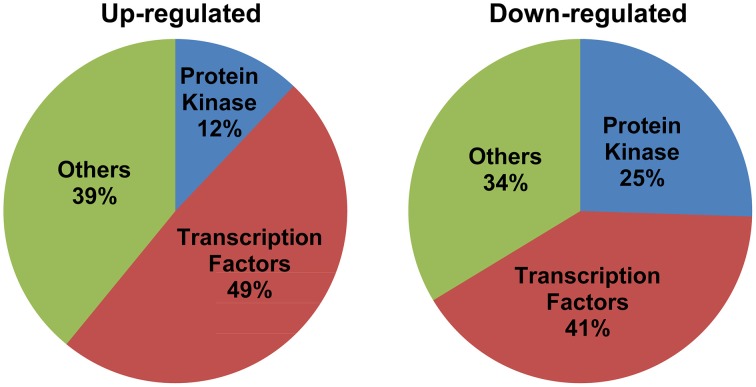
**Signal transduction genes majorly constitute transcription factors and protein kinases**. The genes included in the signal transduction category have been grouped into three classes: transcription factors, protein kinases, and others. Percentage composition of each category has been indicated.

Detailed analysis revealed perturbations in the expression pattern of various transcription factors as well as protein kinases. Also, other signal transduction related genes such as, those involved in hormone signaling, chromatin remodeling, and cell-cell signaling were affected. Importantly, transcription factors constituted a major fraction of MG-responsive signal transduction category, with 49% of the up-regulated and 41% of the down-regulated genes in the signal transduction category being transcription factors (Figure 3). Several transcription factors, such as, bZIP, AP2 domain-containing protein, NAM, WRKY, and zinc finger proteins were found to be induced in response to exogenous MG (Table 1). We could also identify several protein kinases being perturbed in response to MG, with 25% down-regulated and 12% up-regulated genes in the signal transduction category belonging to the kinase family (Figure 3). Various protein kinases such as, MAP kinase (Mitogen-activated protein kinase), calcium/calmodulin-dependent protein kinase (CDPKs), Ser/Thr protein kinase, histidine kinase, and receptor–like kinase showed perturbations in expression. A complete list of protein kinases affected by MG is given in Table 2. Notably, about seven genes of the MAPK pathway were found to be regulated in response to MG. The MAPK superfamily is known to play important roles in response to a variety of cellular stresses (Mizoguchi et al., 1997). In animals and yeast, some reports describe the activation of MAPK pathway even in response to MG (Miyata et al., 2002; Maeta et al., 2005), thereby indicating a role of MAPK pathway in MG-responsive signaling cascade.

**Table 1:** **List of transcription factor genes differentially regulated by methylglyoxal (MG) treatment along with their fold change**.

**Probe ID**	**Accession No**.	**Description**	***p*-value**	**Fold change**
**UP-REGULATED**				
Os.7329.1.S1_s_at	LOC_Os08g42550	AP2 domain containing protein, expressed	0.04	2.58
Os.6367.1.S1_at	LOC_Os01g48060	Auxin response factor, putative, expressed	0.02	2.75
Os.10817.3.S1_x_at	LOC_Os11g32110	Auxin response factor, putative, expressed	0.01	2.28
Os.6367.1.S1_a_at	LOC_Os01g48060	Auxin response factor, putative, expressed	0.05	1.82
OsAffx.26536.1.S1_at	LOC_Os04g49230	B3 DNA binding domain containing protein, expressed	0.05	8.80
OsAffx.15137.1.S1_at	LOC_Os05g47650	B3 DNA binding domain containing protein, expressed	0.03	2.11
OsAffx.25130.1.S1_x_at	LOC_Os03g18210	Basic helix-loop-helix, putative, expressed	0.02	2.54
Os.11132.1.S1_s_at	LOC_Os08g08120	B-box zinc finger family protein, putative, expressed	0.03	4.60
Os.27522.1.S1_x_at	LOC_Os03g58330	BHLH transcription factor, putative, expressed	0.03	1.89
Os.1503.1.S1_at	LOC_Os06g45140	bZIP transcription factor domain containing protein, expressed	0.03	2.35
OsAffx.13332.1.S1_x_at	LOC_Os03g43800	DIRP family protein, putative, expressed	0.02	1.53
Os.54721.1.A1_at	LOC_Os05g51550	DNA binding protein, putative, expressed	0.00	2.50
Os.14023.2.S1_at	LOC_Os06g01370	DNA-binding protein-related, putative, expressed	0.05	2.36
Os.14023.2.S1_x_at	LOC_Os06g01370	DNA-binding protein-related, putative, expressed	0.02	2.30
Os.7574.1.S1_s_at	LOC_Os06g48534	GATA transcription factor 25, putative, expressed	0.02	2.29
OsAffx.24974.1.S1_x_at	LOC_Os03g05160	GATA zinc finger domain containing protein, expressed	0.05	5.97
Os.49626.1.S1_at	LOC_Os03g57260	GRF zinc finger family protein, expressed	0.03	2.37
Os.50790.1.S1_at	LOC_Os03g52320	GRF-interacting factor 1, putative, expressed	0.02	4.14
OsAffx.29777.1.S1_at	LOC_Os09g09460	harpin-induced protein 1 domain containing protein, expressed	0.00	9.11
Os.38400.1.S1_at	LOC_Os09g29830	Helix-loop-helix DNA-binding domain containing protein, expressed	0.05	3.78
OsAffx.6120.1.S1_x_at	LOC_Os08g43070	Helix-loop-helix DNA-binding domain containing protein, expressed	0.05	2.66
Os.14287.1.S1_at	LOC_Os01g64560	Helix-loop-helix DNA-binding protein, putative, expressed	0.03	1.64
OsAffx.23994.1.S1_at	LOC_Os01g70890	Histone-like transcription factor and archaeal histone, putative, expressed	0.03	5.63
Os.49747.1.S1_at	LOC_Os04g55590	Homeobox domain containing protein, expressed	0.02	2.83
Os.35731.1.S1_at	LOC_Os07g38080	Homeodomain-like, putative, expressed	0.04	2.73
Os.13960.2.S1_x_at	LOC_Os01g73560	Leucine rich repeat domain containing protein, putative, expressed	0.04	2.04
Os.19190.3.A1_a_at	LOC_Os03g21230	Leucine-rich repeat family protein, putative, expressed	0.04	1.78
Os.14823.1.S1_at	LOC_Os03g20090	MYB family transcription factor, putative, expressed	0.00	16.99
Os.55708.1.A1_at	LOC_Os02g42870	MYB family transcription factor, putative, expressed	0.03	5.34
Os.55636.1.S1_at	LOC_Os05g48010	MYB family transcription factor, putative, expressed	0.03	3.22
Os.47453.1.A1_s_at	LOC_Os12g39640	MYB family transcription factor, putative, expressed	0.05	2.06
Os.14008.1.S1_at	LOC_Os02g53670	MYB family transcription factor, putative, expressed	0.05	2.03
OsAffx.6404.1.S1_at	LOC_Os09g26170	MYB family transcription factor, putative, expressed	0.03	1.79
Os.52524.1.S1_at	LOC_Os07g48596	Myb transcription factor, putative, expressed	0.04	2.69
OsAffx.2486.1.S1_at	LOC_Os02g02370	Myb-like DNA-binding domain containing protein, putative, expressed	0.04	8.53
OsAffx.24305.1.A1_at	LOC_Os02g18460	NAM, putative, expressed	0.02	2.85
OsAffx.13243.2.S1_at	LOC_Os03g39100	No apical meristem protein, expressed	0.04	2.57
OsAffx.30184.1.S1_at	LOC_Os09g38000	No apical meristem protein, putative, expressed	0.04	2.54
Os.6124.1.S1_a_at	LOC_Os10g21560	No apical meristem protein, putative, expressed	0.01	2.06
OsAffx.23563.1.S1_s_at	LOC_Os01g38780	Nucleic acid binding protein, putative, expressed	0.04	3.44
Os.28015.1.S1_at	LOC_Os01g52680	OsMADS32—MADS-box family gene with MIKCc type-box, expressed	0.02	1.82
Os.4175.1.S1_at	LOC_Os01g69850	OsMADS65—MADS-box family gene with MIKC^*^ type-box, expressed	0.02	1.67
Os.53339.1.S1_a_at	LOC_Os07g49380	PWWP domain containing protein, expressed	0.01	2.62
OsAffx.15318.1.S1_at	LOC_Os06g08820	RING-H2 finger protein, putative, expressed	0.03	2.90
OsAffx.17313.1.S1_at	LOC_Os08g33530	TCP family transcription factor, putative, expressed	0.04	2.17
Os.57558.1.S1_at	LOC_Os04g51000	Transcription factor FL, putative, expressed	0.03	2.23
OsAffx.1957.1.S1_at	LOC_Os01g07880	Transcription factor HY5, putative, expressed	0.04	2.83
Os.6016.1.A1_s_at	LOC_Os08g37810	Transcription factor like protein, putative, expressed	0.04	2.38
OsAffx.11931.1.S1_at	LOC_Os02g07800	Transcription factor, putative, expressed	0.03	5.31
OsAffx.17359.1.S1_at	LOC_Os08g36450	Transcription regulator, putative, expressed	0.03	2.16
Os.50527.1.S1_a_at	LOC_Os03g50110	Transcription regulator, putative, expressed	0.00	2.02
Os.26946.1.S1_at	LOC_Os08g09900	WRKY118, expressed	0.03	3.93
Os.19013.1.S1_at	LOC_Os12g40570	WRKY94, expressed	0.02	2.13
Os.49773.1.S1_at	LOC_Os03g11600	YABBY domain containing protein, putative, expressed	0.01	2.29
Os.11823.1.S1_at	LOC_Os06g41590	YIF1B, putative, expressed	0.03	5.30
Os.15553.1.S1_at	LOC_Os02g06584	Zinc finger C-x8-C-x5-C-x3-H type family protein, expressed	0.04	2.81
OsAffx.13605.1.S1_at	LOC_Os04g01160	Zinc finger family protein, putative, expressed	0.02	4.61
Os.27108.1.S1_at	LOC_Os08g36774	Zinc finger family protein, putative, expressed	0.02	3.40
OsAffx.24591.1.S1_at	LOC_Os02g36000	Zinc finger protein, putative, expressed	0.03	1.90
Os.33152.1.S1_x_at	LOC_Os11g07450	Zinc finger, C3HC4 type domain containing protein, expressed	0.01	2.97
Os.37432.1.S1_at	LOC_Os03g08920	ZINC finger, C3HC4 type domain containing protein, expressed	0.04	2.88
Os.33683.1.S1_at	LOC_Os12g43930	Zinc finger, C3HC4 type domain containing protein, expressed	0.02	2.62
Os.22596.1.S1_s_at	LOC_Os05g28730	Zinc finger, C3HC4 type domain containing protein, expressed	0.02	2.06
OsAffx.26842.1.S1_at	LOC_Os05g11860	Zinc finger, C3HC4 type domain containing protein, expressed	0.02	2.02
Os.7498.1.S1_at	LOC_Os08g42640	Zinc finger, C3HC4 type domain containing protein, expressed	0.05	2.01
OsAffx.17384.1.S1_at	LOC_Os08g37760	Zinc finger, C3HC4 type domain containing protein, expressed	0.05	1.88
Os.212.1.S1_at	LOC_Os01g16950	Zinc finger, C3HC4 type domain containing protein, expressed	0.02	1.70
Os.9372.1.S1_at	LOC_Os07g05610	Zinc ion binding protein, putative, expressed	0.04	1.71
Os.24550.1.S1_at	LOC_Os03g57160	Zinc ion binding protein, putative, expressed	0.05	1.56
OsAffx.11292.1.S1_at	LOC_Os01g32920	ZOS1-08—C2H2 zinc finger protein, expressed	0.05	7.91
OsAffx.23943.1.S1_s_at	LOC_Os01g66570	ZOS1-19—C2H2 zinc finger protein, expressed	0.02	1.65
OsAffx.3743.1.S1_s_at	LOC_Os04g08060	ZOS4-03—C2H2 zinc finger protein, expressed	0.03	1.93
Os.50189.2.S1_at	LOC_Os06g46910	ZOS6-07—C2H2 zinc finger protein, expressed	0.02	7.30
**DOWN-REGULATED**				
OsAffx.11389.1.S1_at	LOC_Os01g39430	Anthocyanin regulatory protein, putative	0.04	3.38
Os.38983.1.S1_at	LOC_Os03g05590	AP2 domain containing protein, expressed	0.05	10.15
Os.30907.1.S1_at	LOC_Os08g45110	AP2 domain containing protein, expressed	0.03	4.25
OsAffx.5131.1.S1_at	LOC_Os06g46995	Armadillo/beta-catenin repeat family protein, putative, expressed	0.01	13.10
Os.5537.1.S1_s_at	LOC_Os09g34880	Basic region leucine zipper domain containing protein, expressed	0.05	2.83
OsAffx.17824.1.S1_at	LOC_Os09g21410	BED zinc finger family protein, expressed	0.03	5.26
OsAffx.27283.2.S1_s_at	LOC_Os01g57580	bHelix-loop-helix transcription factor, putative, expressed	0.03	1.70
OsAffx.11963.1.S1_at	LOC_Os02g09830	bZIP transcription factor domain containing protein, expressed	0.04	7.71
OsAffx.20062.1.S1_at	LOC_Os12g40920	bZIP transcription factor domain containing protein, expressed	0.01	3.36
Os.16422.1.S1_s_at	LOC_Os08g15050	CCT/B-box zinc finger protein, putative, expressed	0.00	3.01
OsAffx.6090.1.S1_at	LOC_Os08g40030	CUP-SHAPED COTYLEDON3, putative, expressed	0.02	5.77
OsAffx.27442.1.S1_at	LOC_Os06g03670	Dehydration-responsive element-binding protein, putative, expressed	0.02	4.16
OsAffx.23629.2.S1_at	LOC_Os01g42050	DNL zinc finger domain containing protein, putative, expressed	0.01	4.78
Os.16203.1.S1_at	LOC_Os01g74590	MYB family transcription factor, putative, expressed	0.00	6.69
OsAffx.14536.1.S1_at	LOC_Os05g04210	MYB family transcription factor, putative, expressed	0.03	1.72
Os.623.2.S1_x_at	LOC_Os01g09640	Myb transcription factor, putative, expressed	0.04	2.10
OsAffx.7264.1.S1_at	LOC_Os11g31360	No apical meristem protein, putative, expressed	0.00	6.10
Os.27469.1.S1_a_at	LOC_Os01g09550	No apical meristem protein, putative, expressed	0.00	5.31
Os.18595.1.A1_at	LOC_Os07g48450	No apical meristem protein, putative, expressed	0.04	2.78
Os.4385.1.S1_at	LOC_Os11g08210	No apical meristem protein, putative, expressed	0.04	2.30
Os.36651.1.S1_at	LOC_Os04g43560	No apical meristem protein, putative, expressed	0.01	1.55
Os.48040.1.A1_at	LOC_Os05g11414	OsMADS58—MADS-box family gene with MIKCc type-box, expressed	0.01	1.70
Os.4153.1.S1_a_at	LOC_Os08g41950	OsMADS7—MADS-box family gene with MIKCc type-box, expressed	0.02	1.88
Os.52097.1.S1_at	LOC_Os04g44440	TCP family transcription factor, putative, expressed	0.04	22.10
Os.15421.1.S1_at	LOC_Os02g55000	Zinc finger CCCH-type with G patch domain-containing protein, putative, expressed	0.01	7.44
Os.55651.1.S1_at	LOC_Os06g11450	Zinc finger, C3HC4 type domain containing protein, expressed	0.02	2.68
Os.19944.1.S1_at	LOC_Os12g43560	Zinc finger, putative, expressed	0.04	1.78
Os.12918.2.S1_x_at	LOC_Os04g58320	Zinc finger, RING-type, putative, expressed	0.03	1.79
Os.49588.1.S1_at	LOC_Os03g41390	ZOS3-15—C2H2 zinc finger protein, expressed	0.04	1.81
Os.15874.1.A1_at	LOC_Os03g60570	ZOS3-22—C2H2 zinc finger protein, expressed	0.05	18.64
Os.56344.1.S1_at	LOC_Os06g20020	ZOS6-03—C2H2 zinc finger protein, expressed	0.04	2.79

**Table 2:** **List of protein kinase genes differentially regulated by methylglyoxal (MG) treatment along with their fold change**.

**Probe ID**	**Accession No**.	**Description**	***p*-value**	**Fold change**
**UP-REGULATED**				
Os.5636.1.S1_at	LOC_Os05g14750	AGC_PVPK_like_kin82y.12—ACG kinases include homologs to PKA, PKG, and PKC, expressed	0.03	8.97
OsAffx.9832.1.S1_x_at	LOC_Os08g27780	OsWAK77—OsWAK receptor-like cytoplasmic kinase OsWAK-RLCK, expressed	0.04	5.70
Os.338.1.S2_a_at	LOC_Os03g17980	CAMK_KIN1/SNF1/Nim1_like_AMPKh.2—CAMK includes calcium/calmodulin depedent protein kinases, expressed	0.00	3.43
Os.338.3.S1_x_at	LOC_Os03g17980	CAMK_KIN1/SNF1/Nim1_like_AMPKh.2—CAMK includes calcium/calmodulin depedent protein kinases, expressed	0.01	2.86
Os.52734.1.S1_at	LOC_Os11g17080	OsMPK15—Putative MAPK based on amino acid sequence homology, expressed	0.05	2.72
OsAffx.30096.1.S1_x_at	LOC_Os09g30190	Receptor-like protein kinase 2 precursor, putative, expressed	0.03	2.43
Os.338.2.S1_x_at	LOC_Os08g37800	CAMK_KIN1/SNF1/Nim1_like_AMPKh.4—CAMK includes calcium/calmodulin depedent protein kinases, expressed	0.01	2.29
Os.436.1.S1_at	LOC_Os01g17250	BRASSINOSTEROID INSENSITIVE 1-associated receptor kinase 1 precursor, putative, expressed	0.04	2.25
Os.17915.1.S1_at	LOC_Os06g17290	Phosphatidylinositol 3- and 4-kinase family protein, putative, expressed	0.01	2.24
Os.17824.1.S1_at	LOC_Os02g42620	Protein kinase, putative, expressed	0.05	2.22
Os.26917.1.A1_at	LOC_Os02g47220	CPuORF20—conserved peptide uORF-containing transcript, expressed	0.01	2.18
Os.3356.1.S1_at	LOC_Os10g25090	STRUBBELIG-RECEPTOR FAMILY 6 precursor, putative, expressed	0.05	2.18
Os.8740.1.S1_at	LOC_Os02g14530	Protein kinase domain containing protein, expressed	0.05	2.05
Os.56163.1.S1_at	LOC_Os04g04000	ECT protein, putative, expressed	0.01	2.03
Os.49462.1.S1_at	LOC_Os02g44642	STE_MEKK_ste11_MAP3K.10—STE kinases include homologs to sterile 7, sterile 11, and sterile 20 from yeast, expressed	0.00	1.89
Os.52830.1.S1_at	LOC_Os02g34430	Serine/threonine-protein kinase, putative, expressed	0.04	1.86
Os.11626.1.S1_at	LOC_Os04g56530	STE_MEKK_ste11_MAP3K.1—STE kinases include homologs to sterile 7, sterile 11, and sterile 20 from yeast, expressed	0.01	1.80
Os.19371.1.S1_at	LOC_Os12g29580	AGC_PVPK_like_kin82y.19—ACG kinases include homologs to PKA, PKG, and PKC, expressed	0.01	1.72
Os.23942.1.A1_at	LOC_Os02g35180	OsRR2 type-A response regulator, expressed	0.02	1.65
Os.20399.1.S1_s_at	LOC_Os01g62080	Serine/threonine-protein kinase AFC1, putative, expressed	0.03	1.63
Os.12648.1.S1_at	LOC_Os09g08420	AGC_PVPK_like_kin82y.15—ACG kinases include homologs to PKA, PKG, and PKC, expressed	0.01	1.63
**DOWN-REGULATED**				
OsAffx.15056.1.S1_s_at	LOC_Os05g41270	CAMK_CAMK_like.4—CAMK includes calcium/calmodulin depedent protein kinases, expressed	0.05	5.97
Os.10274.4.S1_at	LOC_Os12g03810	CAMK_KIN1/SNF1/Nim1_like.37—CAMK includes calcium/calmodulin depedent protein kinases, expressed	0.02	3.87
Os.55776.1.S1_x_at	LOC_Os01g06876	Cf-2, putative, expressed	0.02	2.55
Os.14999.3.S1_x_at	LOC_Os01g47530	CGMC_MAPKCMGC_2.6—CGMC includes CDA, MAPK, GSK3, and CLKC kinases, expressed	0.02	2.12
OsAffx.10273.1.S1_at	LOC_Os07g03970	Lectin-like receptor kinase 7, putative, expressed	0.01	23.36
OsAffx.29778.1.S1_at	LOC_Os09g09500	Lectin-like receptor kinase, putative, expressed	0.01	6.57
Os.12660.1.S1_at	LOC_Os07g38800	Lectin-like receptor kinase, putative, expressed	0.02	4.74
Os.50228.1.S1_at	LOC_Os07g15940	Legume lectins beta domain containing protein, expressed	0.05	11.24
Os.29772.1.S1_at	LOC_Os03g40250	Leucine Rich Repeat family protein, expressed	0.03	2.72
OsAffx.31317.1.S1_at	LOC_Os11g35960	Leucine rich repeat N-terminal domain containing protein, putative, expressed	0.05	3.69
OsAffx.27458.1.S1_at	LOC_Os06g04810	Leucine rich repeat protein, putative, expressed	0.03	1.51
Os.50342.1.S1_at	LOC_Os04g52780	Leucine-rich repeat receptor protein kinase EXS precursor, putative, expressed	0.02	1.82
OsAffx.20258.1.S1_at	LOC_Os10g33080	Leucine-rich repeat receptor protein kinase EXS precursor, putative, expressed	0.03	2.55
OsAffx.18277.1.S1_at	LOC_Os10g17910	OsWAK114—OsWAK receptor-like cytoplasmic kinase OsWAK-RLCK, expressed	0.00	7.20
Os.19575.1.A1_at	LOC_Os04g30010	OsWAK45—OsWAK receptor-like protein kinase, expressed	0.02	4.60
Os.14874.2.S1_at	LOC_Os01g47430	Protein of unknown function DUF1296 domain containing protein, expressed	0.01	5.02
OsAffx.7310.1.S1_at	LOC_Os11g36090	Receptor kinase, putative, expressed	0.01	1.75
Os.46331.1.S1_s_at	LOC_Os01g66040	Receptor kinase, putative, expressed	0.02	3.15
Os.49804.1.S1_at	LOC_Os11g47310	Receptor kinase-like protein, identical, putative, expressed	0.03	1.88
Os.27053.2.S1_s_at	LOC_Os11g07200	Receptor protein kinase CLAVATA1 precursor, putative, expressed	0.04	1.95
OsAffx.12352.1.S1_at	LOC_Os02g34770	Receptor-like protein kinase 2 precursor, putative	0.01	2.49
Os.11775.1.S1_a_at	LOC_Os01g53920	Receptor-like protein kinase 5 precursor, putative, expressed	0.01	7.95
Os.27084.1.A2_at	LOC_Os04g52640	SHR5-receptor-like kinase, putative, expressed	0.01	2.10
Os.49145.1.S1_at	LOC_Os03g27990	STRUBBELIG-RECEPTOR FAMILY 7 precursor, putative, expressed	0.00	2.91
Os.26816.1.A1_s_at	LOC_Os07g35410	TKL_IRAK_DUF26-lc.18—DUF26 kinases have homology to DUF26 containing loci, expressed	0.01	2.20

Another important observation was the effect of elevated MG levels on the expression of stress-responsive components of the signal transduction machinery. Transcript levels of several stress-inducible transcription factors like DREB, MYB, NAC, WRKY, and AP2 domain containing proteins, and protein kinases such as OsRR2 type-A response regulator, were altered upon MG exposure (Tables 1, 2). In case of MYB family transcription factors, 8 different genes were up-regulated and three were down-regulated following MG treatment. Likewise, nine NAM genes, eight zinc finger C_3_HC_4_ type domain-containing protein and seven C_2_H_2_ zinc finger protein encoding genes were found to be differentially regulated after MG application (Table 1). Since MG levels increase under various abiotic and biotic stress conditions, it is now believed to be a common consequence of stress and thus, it is not surprising to observe a change in expression of the above mentioned stress-inducible transcription factors and protein kinases upon MG treatment. Also, it is very likely that MG-responsive transcriptome significantly overlaps with the abiotic stress-responsive transcriptome as we could find various stress-inducible genes being affected by MG.

### Identification of conserved motifs in the upstream region of MG-responsive genes

Being a potent glycating agent, MG can readily modify DNA, protein, and phospholipid moieties. Importantly, DNA is susceptible to irreversible modifications by MG, with deoxyguanosine being the most reactive nucleotide under physiological conditions (Thornalley, 2008). The ability of MG to directly react with DNA prompted us to identify conserved motifs in the 1 kb upstream region of MG-responsive genes which can potentially act as MG-responsive elements (MGREs). For this purpose, genes whose expression was altered (either up- or down-regulated) more than 10-fold in response to MG were selected for analysis.

**Figure 4:**
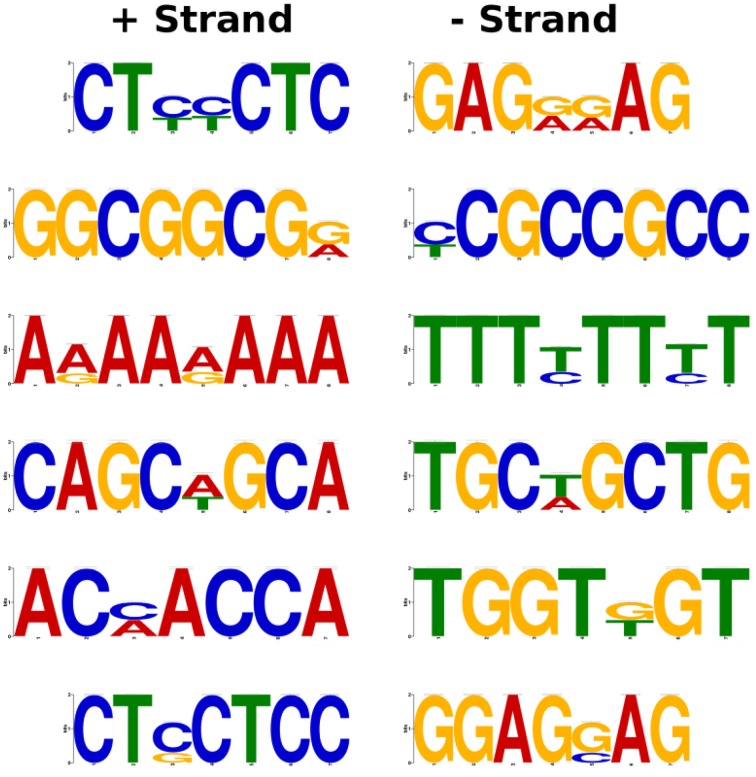
**Prediction of putative MG-responsive elements (MGREs) as conserved motifs in the promoter region of MG-responsive genes**. The genes with more than 10-fold alteration in expression upon MG treatment were selected for analysis. The 1 kb upstream region of MG-responsive genes was used for the identification of motifs using MEME software.

We retrieved the upstream sequences of around 160 genes being more than 10-fold altered in expression upon MG treatment including the promoter sequences of the members of the glyoxalase family as well. Screening of the upstream region using Multiple Expectation for Motif Elicitation (MEME) software predicted the presence of 7–8 bp long conserved motifs (Figure 4). Most of the predicted motifs were found to be C/G rich regions, like CTXXCTC and GGCGGCGX. Though, as an exception an all A/T rich motif was also observed during the motif search. The results of the *in silico* analysis however, need to be validated through biological experiments. Nevertheless, the data gives some indication toward the presence of MG-responsive elements which regulate gene expression upon perception of increasing MG levels.

## Discussion

Despite the recognized toxic nature of MG in living systems, little is known about the molecular mechanisms determining MG response and subsequent growth arrest in plants. In the present study, we have demonstrated the toxic effects of MG on growth of rice seedlings followed by a microarray-based expression profiling study to investigate changes in the gene expression patterns upon exogenous MG application.

MG affected the growth of rice seedlings in a dose-dependent manner similar to that observed in Arabidopsis (Hoque et al., 2012), with severe retardation at concentrations higher than 10 mM MG. Analysis of gene expression patterns revealed large-scale perturbations in the rice transcriptome upon exposure to 10 mM MG concentration affecting diverse biological processes such as, metabolism, transport, signal transduction, transcription, and translation. A similar MG-mediated genome-wide alteration in expression profile has been earlier reported in human endothelial cells as well (Lee et al., 2011). MG-derived modifications can be either through direct interaction with DNA and/or RNA or indirectly by modifying the activity of proteins involved in diverse biological pathways (Thornalley, 2008).

**Figure 5:**
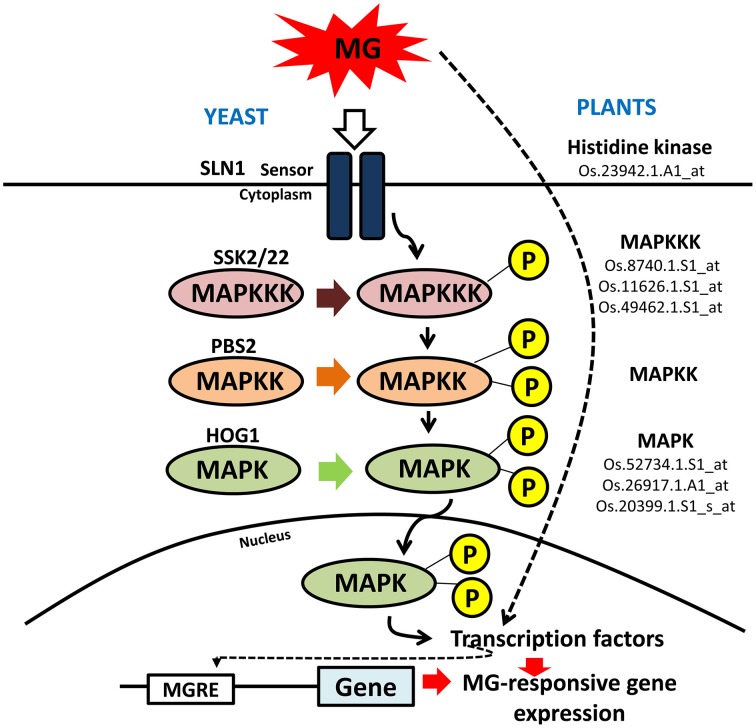
**Schematic representation of MAPK signaling cascade in yeast and plants**. Mitogen-activated protein kinase (MAPK) signaling cascade is triggered in yeast in response to MG. A similar pathway is proposed to operate in rice as well in response to MG. The rice counterparts found to be induced by MG application have been shown along with their probe IDs. MG can modify expression of genes either by inducing the signal cascade through sensor proteins or it can directly affect the activity of transcription factor that goes and binds the promoter region at MG-responsive elements (MGRE) and regulate gene expression.

What is more interesting is the strong representation of genes involved in signal transduction pathway. MG, like nitric oxide (NO) and hydrogen peroxide (H_2_O_2_) perturbs a significant proportion of signaling genes. NO and H_2_O_2_ are important signaling molecules and in fact, many similarities can be drawn between the two related to transcriptional responses induced by them, with both exhibiting significant cross-talk with general stress-responsive pathways (Neill et al., 2002). Like NO and H_2_O_2_, MG could induce the transcript levels of various protein kinases and transcription factors, with many known to be involved in abiotic stress response. MG is probably perceived as a stress signal by the system through some sensor proteins, as happens in yeast through Sln1 (Maeta et al., 2005) and subsequently signals the activation of several components of signaling pathways. For MG to really act as a specific signaling molecule, mechanisms must exist to sense elevation in its levels in cells, which is possible through MG-mediated reversible modification at cysteine residues within proteins (Thornalley, 2008). This redox modulation in turn can alter the protein conformation and thereby triggering a cellular response. For example, MG is known to modify Akt1/PKB, a protein kinase, at Cys residue in L6 muscle cells thereby increasing its phosphorylation and hence affecting its activity; subsequently initiating downstream cellular responses (Chang et al., 2011). In yeast, role of MG as a signal initiator is well-defined, activating the Hog1-MAP kinase cascade through Sln1 branch (Maeta et al., 2005). Sln1 is an osmosensor with histidine kinase activity that functions as a sensor of MG. Upon MG stimulation, Hog1 is phosphorylated by Pbs2 and translocated into the nucleus. The nuclear Hog1 recruits transcription factors (such as, Msn2 and Msn4) to the promoter region, thereby activating transcription of the genes under its control, including MG-detoxifying GLYI (Maeta et al., 2005). In addition, MG also stimulates Yap1, a bZIP transcription factor that is predominantly distributed in the cytoplasm under normal conditions in yeast but upon MG stimulation translocates to the nucleus and functions in regulating gene expression (Maeta et al., 2004). In plants, little to nothing is known regarding the role of MG in signaling. Based on the existing reports in animals and yeast (reviewed by Kaur et al., 2014a), we suggest that even in plants similar MAPK pathway might be involved in MG signaling (Figure 5). In agreement, we observed an induction in the transcript levels of a putative histidine kinase gene and six MAPK genes (mentioned in Figure 5). In plants, MAPKs are usually known to be activated in response to various environmental signals such as drought, cold, osmotic stress, and pathogen challenge (Mizoguchi et al., 1997). In fact, MG has also been shown to induce the p38/MAPK pathway in animals, regulating various cellular processes (reviewed by Kaur et al., 2014a). The MAPK cascade is now considered as a central pathway mediating tolerance to various stresses (Smékalová et al., 2014), and may be to MG stress also. Further, even in response to NO and H_2_O_2_, MAPK cascade is believed to be the focal point of signal transmission and also convergence with various stress-inducible signaling pathways (Neill et al., 2002).

Since MG and abiotic stresses are inextricably linked (Yadav et al., 2005a; Kaur et al., 2014b), we could detect various stress-inducible transcription factors to be induced upon MG application. For instance, several NAC genes were found to be up-regulated which can bind to the promoter region of stress-responsive genes including glyoxalase and promote their transcription in order to lower MG levels and associated stress (Fujita et al., 2004). The WRKY transcription factors also have well-established roles in abiotic and biotic stress response and even participate in several developmental and physiological processes, leaf senescence, regulation of biosynthetic pathways and hormone signaling (Chen et al., 2012). Similarly, C_2_H_2_-type zinc finger protein genes are known to activate stress-related genes and enhance tolerance to salt, dehydration, and/or cold stresses (Sun et al., 2010; Kiełbowicz-Matuk, 2012). A cell wall-associated receptor-like kinase (OsWAK77) was also up-regulated. WAK genes are involved in various functions in plants, including pathogen resistance (He et al., 1998), abiotic stress response (Gao and Xue, 2012), heavy-metal tolerance (Sivaguru et al., 2003), and plant development (Lally et al., 2001). Additionally, MG was also found to induce the expression of genes involved in metabolic signaling, such as, a SnRK1-type of kinase, which encodes an energy sensor protein that regulates gene expression in response to energy depletion in plants (Cho et al., 2012). Furthermore, our motif search predicted 7–8 bp long conserved elements in the promoter region of genes showing more than 10-fold change in expression in the microarray analysis that may serve as possible MG-responsive element (MGRE).

**Figure 6:**
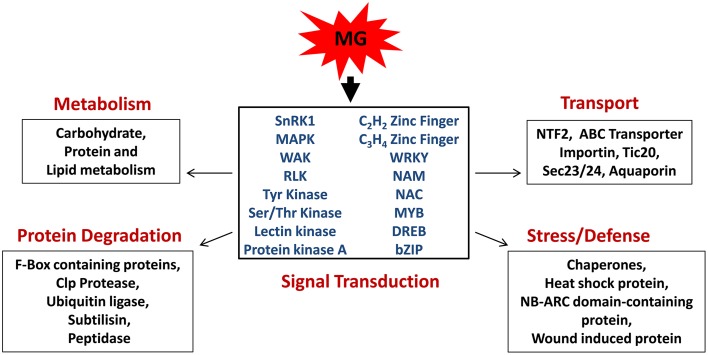
**Model depicting the role of methylglyoxal as a signaling molecule in rice**. MG induces the expression of signal transduction genes, mainly protein kinases, and transcription factors, which subsequently regulate various cellular processes, such as metabolism, transport, stress, and defense response, and protein degradation.

Taken together, through this study we provide first report on the effect of MG on global gene expression profile of rice. We propose that plants perceive MG as a stress signal and trigger a response via induction of several protein kinases and transcription factors which then affect the expression of downstream targets involved in various biological pathways, thereby causing a global change in plant transcriptome (Figure 6). In addition, other mode of action may also exist, involving direct modification of effector proteins by MG, thereby regulating their expression and activity.

## Author contributions

SS conceived the idea and designed the experiments. CK, HK, and AM performed the analysis and wrote the manuscript. SLS, AP, and SKS edited the manuscript. All the authors approved the final manuscript.

### Conflict of interest statement

The guest associate editor Girdhar K. Pandey declares that, despite being having collaborated in the past with the authors Sudhir K. Sopory and Sneh L. Singla-Pareek, the review process was handled objectively. The authors declare that the research was conducted in the absence of any commercial or financial relationships that could be construed as a potential conflict of interest.

## References

[B1] Bailey T. L. Boden M. Buske F. A. Frith M. Grant C. E. Clementi L.. (2009). MEME SUITE: tools for motif discovery and searching. Nucleic Acids Res. 37, W202–W208. 10.1093/nar/gkp335 19458158 PMC2703892

[B2] Booth I. R. Ferguson G. P. Miller S. Li C. Gunasekera B. Kinghorn S. (2003). Bacterial production of methylglyoxal: a survival strategy or death by misadventure? Biochem. Soc. Trans. 31, 1406–1408. 10.1042/bst0311406 14641075

[B3] Campbell A. K. Naseem R. Holland I. B. Matthews S. B. Wann K. T. (2007). Methylglyoxal and other carbohydrate metabolites induce lanthanum-sensitive Ca^2+^ transients and inhibit growth in *E.* coli. Arch. Biochem. Biophys. 468, 107–113. 10.1016/j.abb.2007.09.006 17961498

[B4] Chandler P. Robertson M. (1994). Gene expression regulated by abscisic acid and its relation to stress tolerance. Ann. Rev. Plant Physiol. Plant Mol. Biol. 45, 113–141. 10.1146/annurev.pp.45.060194.000553

[B5] Chang T. Wang R. Olson D. J. Mousseau D. D. Ross A. R. Wu L. (2011). Modification of Akt1 by methylglyoxal promotes the proliferation of vascular smooth muscle cells. FASEB J. 25, 1746–1757. 10.1096/fj.10-178053 21321187

[B6] Chen L. Song Y. Li S. Zhang L. Zou C. Yu D. (2012). The role of WRKY transcription factors in plant abiotic stresses. Biochim. Biophys. Acta 1819, 120–128. 10.1016/j.bbagrm.2011.09.002 21964328

[B7] Cho Y. H. Hong J. W. Kim E. C. Yoo S. D. (2012). Regulatory functions of SnRK1 in stress-responsive gene expression and in plant growth and development. Plant Physiol. 158, 1955–1964. 10.1104/pp.111.189829 22232383 PMC3320198

[B8] Ferguson G. P. Totemeyer S. MacLean M. J. Booth I. R. (1998). Methylglyoxal production in bacteria: suicide or survival? Arch. Microbiol. 170, 209–218. 10.1007/s002030050635 9732434

[B9] Fujita M. Fujita Y. Maruyama K. Seki M. Hiratsu K. Ohme-Takagi M.. (2004). A dehydration-induced NAC protein, RD26, is involved in a novel ABA-dependent stress-signaling pathway. Plant J. 39, 863–876. 10.1111/j.1365-313X.2004.02171.x 15341629

[B10] Gao L. L. Xue H. W. (2012). Global analysis of expression profiles of rice receptor-like kinase genes. Mol. Plant 5, 143–153. 10.1093/mp/ssr062 21765177

[B11] Ghosh A. Pareek A. Sopory S. K. Singla-Pareek S. L. (2014). A glutathione responsive rice glyoxalase II, OsGLYII-2, functions in salinity adaptation by maintaining better photosynthesis efficiency and anti-oxidant pool. Plant J. 80, 93–105. 10.1111/tpj.12621 25039836

[B12] He Z. H. He D. Kohorn B. D. (1998). Requirement for the induced expression of a cell wall associated receptor kinase for survival during the pathogen response. Plant J. 14, 55–63. 10.1046/j.1365-313X.1998.00092.x 9681026

[B13] Hoque T. S. Uraji M. Tuya A. Nakamura Y. Murata Y. (2012). Methylglyoxal inhibits seed germination and root elongation and up-regulates transcription of stress-responsive genes in ABA-dependent pathway in Arabidopsis. Plant Biol. (Stuttg) 14, 854–858. 10.1111/j.1438-8677.2012.00607.x 22676051

[B14] Hossain M. A. Hossain M. Z. Fujita M. (2009). Stress- induced changes of methylglyoxal level and glyoxalase. I activity in pumpkin seedlings and cDNA cloning of glyoxalase I gene. Aust. J. Crop Sci. 3, 53–64.

[B15] Kalapos M. P. (1999). Methylglyoxal in living organisms: chemistry, biochemistry, toxicology and biological implications. Toxicol. Lett. 110, 145–175. 10.1016/S0378-4274(99)00160-5 10597025

[B16] Kaur C. Ghosh A. Pareek A. Sopory S. K. Singla-Pareek S. L. (2014b). Glyoxalases and stress tolerance in plants. Biochem. Soc. Trans. 42, 485–490. 10.1042/BST20130242 24646265

[B17] Kaur C. Singla-Pareek S. L. Sopory S. K. (2014a). Glyoxalase and methylglyoxal as biomarkers for plant stress tolerance. Crit. Rev. Plant Sci. 33, 429–456. 10.1080/07352689.2014.904147

[B18] Kaur C. Vishnoi A. Ariyadasa T. U. Bhattacharya A. Singla-Pareek S. L. Sopory S. K. (2013). Episodes of horizontal gene-transfer and genefusion led to co-existence of different metal-ion specific glyoxalase I. Sci. Rep. 3, 3076. 10.1038/srep03076 24220130 PMC3826101

[B19] Kiełbowicz-Matuk A. (2012). Involvement of plant C_2_H_2_-typezincfinger transcription factors in stress responses. Plant Sci. 185–186, 78–85. 10.1016/j.plantsci.2011.11.015 22325868

[B20] Lally D. Ingmire P. Tong H. Y. He Z. H. (2001). Antisense expression of a cell wall-associated protein kinase, WAK4, inhibits cell elongation and alters morphology. Plant Cell 13, 1317–1331. 10.1105/tpc.13.6.1317 11402163 PMC135583

[B21] Lee S. E. Yang H. Jeong S. I. Jin Y. H. Park C. S. Park Y. S. (2011). Methylglyoxal-mediated alteration of gene expression in human endothelial cells. Biochip J. 5, 220–228. 10.1007/s13206-011-5305-y

[B22] Maeta K. Izawa S. Inoue Y. (2005). Methylglyoxal, a metabolite derived from glycolysis, functions as a signal initiator of the high osmolarity glycerol-mitogen-activated protein kinase cascade and calcineurin/Crz1-mediated pathway in Saccharomyces cerevisiae. J. Biol. Chem. 280, 253–260. 10.1074/jbc.M408061200 15520007

[B23] Maeta K. Izawa S. Okazaki S. Kuge S. Inoue Y. (2004). Activity of the Yap1 transcription factor in *Saccharomyces cerevisiae* is modulated by methylglyoxal, a metabolite derived from glycolysis. Mol. Cell Biol. 24, 8753–8764. 10.1128/MCB.24.19.8753-8764.2004 15367692 PMC516737

[B24] Miyata S. Miyazaki H. Liu B. F. Fukunaga M. Hamada Y. Ueyama S. (2002). Activation of MAP kinase superfamily signaling pathways by methylglyoxal. Int. Congr. Ser. 1245, 87–89. 10.1016/s0531-5131(02)00913-5

[B25] Mizoguchi T. Ichimura K. Shinozaki K. (1997). Environmental stress response in plants: the role of mitogen-activated protein kinases. Trends Biotechnol. 15, 15–19. 10.1016/S0167-7799(96)10074-3 9032988

[B26] Mukhopadhyay P. Reddy M. K. Singla-Pareek S. L. Sopory S. K. (2011). Transcriptional downregulation of rice rpL32 gene under abiotic stress is associated with removal of transcription factors within the promoter region. PLoS ONE 6:e28058. 10.1371/journal.pone.0028058 22132208 PMC3223225

[B27] Mustafiz A. Singh A. K. Pareek A. Sopory S. K. Singla-Pareek S. L. (2011). Genome-wide analysis of rice and Arabidopsis identifies two glyoxalase genes that are highly expressed in abiotic stresses. Funct. Integr. Genomics 11, 293–305. 10.1007/s10142-010-0203-2 21213008

[B28] Neill S. J. Desikan R. Clarke A. Hurst R. D. Hancock J. T. (2002). Hydrogen peroxide and nitric oxide as signaling molecules in plants. J. Exp. Bot. 53, 1237–1247. 10.1093/jexbot/53.372.1237 11997372

[B29] Rabbani N. Thornalley P. J. (2012). Methylglyoxal, glyoxalase 1 and the dicarbonyl proteome. Amino Acids 42, 1133–1142. 10.1007/s00726-010-0783-0 20963454

[B30] Rabbani N. Thornalley P. J. (2014). Dicarbonyl proteome and genome damage in metabolic and vascular disease. Biochem. Soc. Trans. 42, 425–432. 10.1042/BST20140018 24646255

[B31] Ramasamy R. Yan S. F. Schmidt A. M. (2006). Methylglyoxal comes of AGE. Cell 124, 258–260. 10.1016/j.cell.2006.01.002 16439200

[B32] Ray S. Dutta S. Halder J. Ray M. (1994). Inhibition of electron flow through complex I of the mitochondrial respiratory chain of Ehrlich ascites carcinoma cells by methylglyoxal. Biochem. J. 303, 69–72. 10.1042/bj3030069 7945267 PMC1137558

[B33] Singla-Pareek S. L. Reddy M. K. Sopory S. K. (2003). Genetic engineering of the glyoxalase pathway in tobacco leads to enhanced salinity tolerance. Proc. Natl. Acad. Sci. U.S.A. 100, 14672–14677. 10.1073/pnas.2034667100 14638937 PMC299757

[B34] Singla-Pareek S. L. Yadav S. K. Pareek A. Reddy M. K. Sopory S. K. (2006). Transgenic tobacco overexpressing glyoxalase pathway enzymes grow and set viable seeds in zinc-spiked soils. Plant Physiol. 140, 613–623. 10.1104/pp.105.073734 16384901 PMC1361328

[B35] Sivaguru M. Ezaki B. He Z. H. Tong H. Osawa H. Baluska F.. (2003). Aluminum-induced gene expression and protein localization of a cell wall-associated receptor kinase in Arabidopsis. Plant Physiol. 132, 2256–2266. 10.1104/pp.103.022129 12913180 PMC181309

[B36] Smékalová V. Doskočilová A. Komis G. Samaj J. (2014). Crosstalk between secondary messengers, hormones and MAPK modules during abiotic stress signalling in plants. Biotechnol. Adv. 32, 2–11. 10.1016/j.biotechadv.2013.07.009 23911976

[B37] Sun S. J. Guo S. Q. Yang X. Bao Y. M. Tang H. J. Sun H.. (2010). Functional analysis of a novel Cys2/His2-type zinc finger protein involved in salt tolerance in rice. J. Exp. Bot. 61, 2807–2818. 10.1093/jxb/erq120 20460361 PMC2882275

[B38] Thornalley P. J. (2008). Protein and nucleotide damage by glyoxal and methylglyoxal in physiological systems—role in ageing and disease. Drug Metabol. Drug Interact. 23, 125–150. 10.1515/DMDI.2008.23.1-2.125 18533367 PMC2649415

[B39] Yadav S. K. Singla-Pareek S. L. Ray M. Reddy M. K. Sopory S. K. (2005a). Methylglyoxal levels in plants under salinity stress are dependent on glyoxalase I and glutathione. Biochem. Biophys. Res. Commun. 337, 61–67. 10.1016/j.bbrc.2005.08.263 16176800

[B40] Yadav S. K. Singla-Pareek S. L. Reddy M. K. Sopory S. K. (2005b). Transgenic tobacco plants overexpressing glyoxalase enzymes resist an increase in methylglyoxal and maintain higher reduced glutathione levels under salinity stress. FEBS Lett. 579, 6265–6271. 10.1016/j.febslet.2005.10.006 16253241

[B41] Yoshiba Y. Kiyosue T. Nakashima K. Yamaguchi-Shinozaki K. Shinozaki K. (1997). Regulation of levels of proline as an osmolyte in plants under water stress. Plant Cell Physiol. 38, 1095–1102. 10.1093/oxfordjournals.pcp.a029093 9399433

[B42] Yoshida S. Forno D. A. Cock J. H. Gomez K. A. (1972). Laboratory Manual for Physiological Studies of Rice. Manila: International Rice Research Institute.

