# Supplementary material for: Analysis of global gene expression profile of rice in response to methylglyoxal indicates its possible role as a stress signal molecule
Source: Front Plant Sci. 2015 Sep 3;6:682. doi: 10.3389/fpls.2015.00682 (PMC4558467; doi:10.3389/fpls.2015.00682)
Supplement: Supplementary file 1 [file Table1.PDF]

Table S1. Table showing correlation coefficient across arrays. The coefficients were calculated using Pearson Correlation Coefficient.

Array name	C1	C2	MG1	MG2	Group
C1	1	0.933142	0.845525	0.458043	Control
C2	0.933142	1	0.928965	0.53701	Control
MG1	0.845525	0.928965	1	0.619415	MG
MG2	0.458043	0.53701	0.619415	1	MG
