# Supplementary material for: Analysis of global gene expression profile of rice in response to methylglyoxal indicates its possible role as a stress signal molecule
Source: Front Plant Sci. 2015 Sep 3;6:682. doi: 10.3389/fpls.2015.00682 (PMC4558467; doi:10.3389/fpls.2015.00682)

**Figure S2**. Unsupervised hierarchical clustering of differentially expressed genes in the four sample arrays.

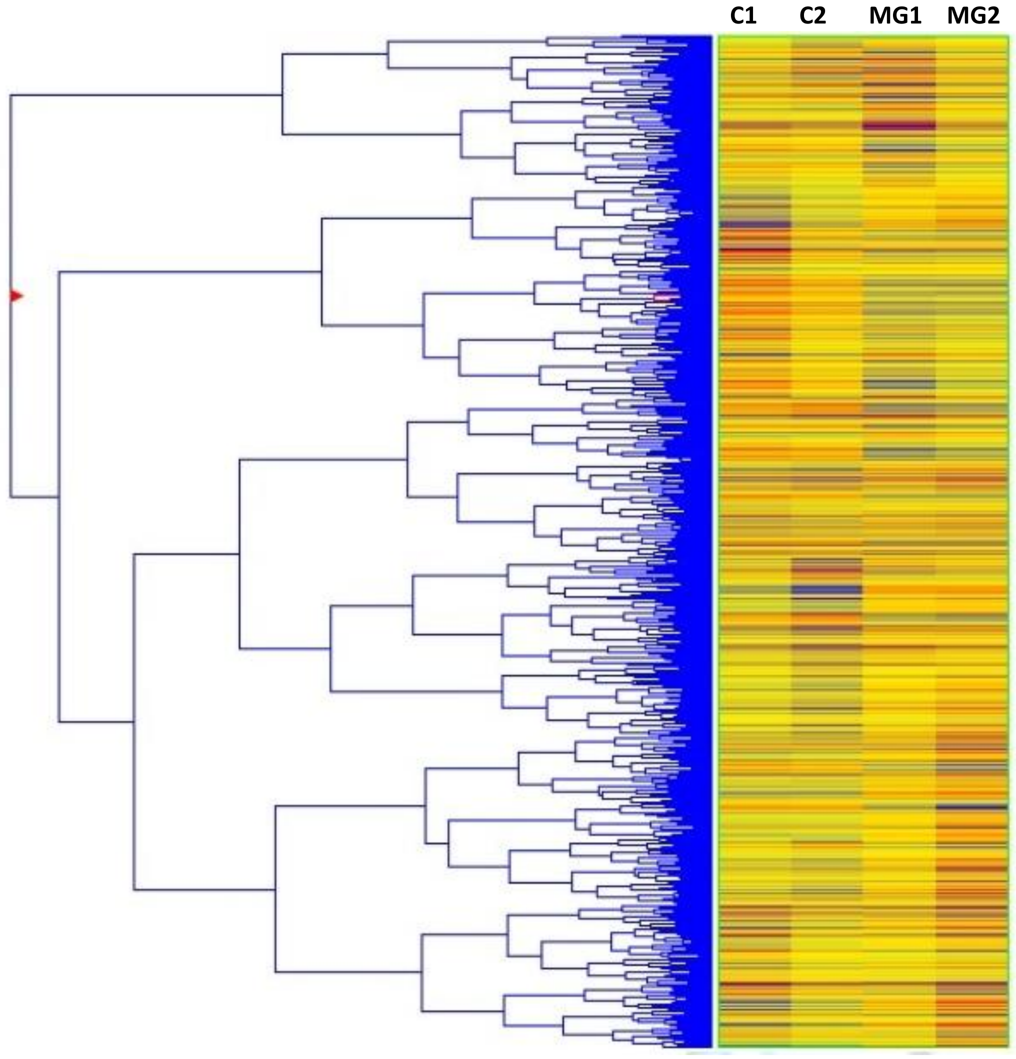

Supplement: Supplementary file 3 [file Presentation2.PDF]
